# Illinois Medical Journal

**Published:** 1844-06

**Authors:** 


### ILLINOIS MEDICAL JOURNAL.

A new journal is offered to the profession from the far west. Dr. Blaney, of Chicago, issued the first number of the *Illinois Medical and Surgical Journal* in April, and proposes to publish it monthly at $1 per annum. At this price, it would be supposed that any physician might afford to take it. The Illinois Medical Journal prefers some peculiar claims upon the profession in that thriving quarter of our country. In the nature of the case, it must possess a local interest, and it will afford to the physicians in that region a medium through which they may make known their views and experience. The medical schools in the United States sent out at the late commencements, we believe, twelve hundred graduates, who are now assuming the responsibility of their profession, and who ought to be reading men. Such accessions to the ranks of the profession must multiply rapidly the subscribers to medical journals, and in the growth, room, it is to be hoped, will be found for the new journal of Illinois. Y

